# Cutaneous sarcoid‐like reaction in a patient treated with target therapy for metastatic melanoma: the hue is the clue

**DOI:** 10.1111/dth.15731

**Published:** 2022-08-02

**Authors:** Maria Francesca Baracca, Martina Lambertini, Lidia Sacchelli, Cosimo Misciali, Barbara Melotti, Carlotta Gurioli, Emi Dika

**Affiliations:** ^1^ Dermatology Unit IRCCS Azienda Ospedaliero‐Universitaria di Bologna Bologna Italy; ^2^ Division of Dermatology, Department of Experimental, Diagnostic and Specialty Medicine (DIMES) University of Bologna Bologna Italy; ^3^ Division of Oncology IRCCS Azienda Ospedaliero‐Universitaria di Bologna Bologna Italy

Dear Editor,

A 54‐year‐old Caucasian woman had been undergoing regular follow‐up at our Melanoma service for a previous nodular melanoma of the dorsum (Breslow thickness 3.6 mm with ulceration). The molecular analysis had shown a *BRAF V600E* mutation. The patient was in adjuvant treatment with dabrafenib + trametinib for the previous detection of lymphonodal metastasis. After 11 months from the beginning of the therapy, she reported the development of two bilateral skin lesions on the crease of her elbows in proximity of the intravenous drug injection sites. At clinical examination firm, infiltrated, asymptomatic purplish papules, and plaques were detected (Figure 1A,B). The patient did not refer any other drug intake. Dermoscopy revealed polymorphous vessels (hairpin, linear, and comma) on an erythematous to the orange–yellow background (Figure 1C). A biopsy was performed and histology showed a granulomatous sarcoid‐like infiltrate (non‐necrotizing dermal granulomas with epithelioid and multinucleated giant cells). Grocott and Ziehl–Neelsen staining resulted negative. The patient had no extracutaneous symptoms (uveitis, clinical lymphadenopathy, bilateral hilar lymphadenopathy, and pulmonary involvement), nor mucosal involvement. No signs and symptoms of arthritis or bone involvement were found. Blood tests were within normal ranges, including PCR, erythrocyte sedimentation rate, liver function, serum calcium level, protein electrophoresis, and ACE level. Urinary protein level, quantiFERON‐TB tests, and thoracic scans were negative. The oncological condition was stable and in remission at the time of the sarcoid‐like reaction (SLR) onset. Due to the lack of systemic signs a final diagnosis of SLR related to *BRAF* inhibitor therapy was performed. The patient was treated with clobetasol cream with benefit.

**FIGURE 1:**
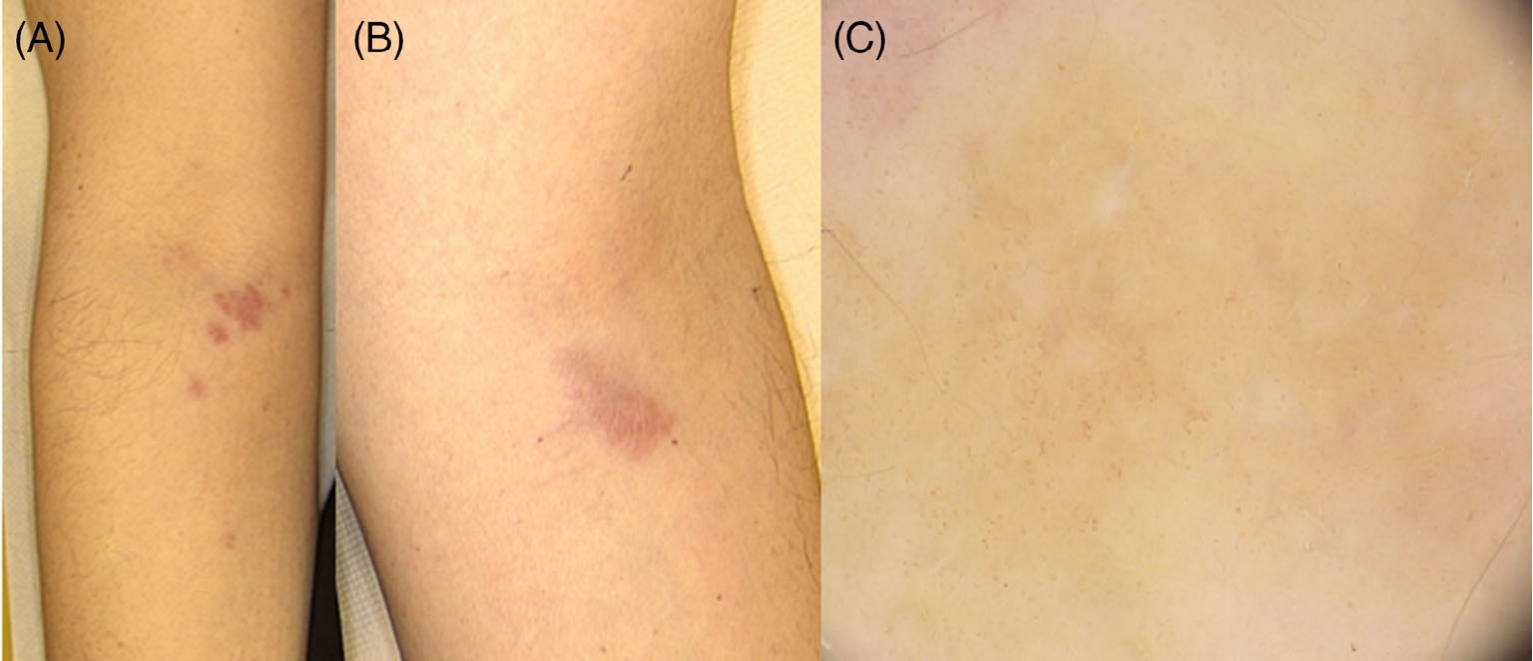
Clinical presentation (A) and (B) showing firm, infiltrated, asymptomatic purplish papules and plaques on the crease of the elbows. Dermoscopy (C) reveals polymorphous vessels (hairpin, linear, and comma) on an erythematous to orange–yellow background

*BRAF* inhibitors have been reported to induce granulomatous reactions, including sarcoidosis and SLR.^1^ These adverse conditions are infrequent, with a prevalence of 0.58% in a cohort of melanoma patients.^2^ SLR may even occur as a paraneoplastic phenomenon.^3^

Sarcoidosis is a multisystem granulomatous disease of unknown cause characterized by an altered immune activation. The key cells that initiate the granulomatous reaction are T‐helper 1 cells that produce interleukin 2 and interferon (IFN)‐γ, causing an overactivation of macrophages and T cells and also leading to the increased secretion of TNFα. The consequence of this hyperactivation is the formation of granulomas with epithelioid histocytes and giant cells.^4^, ^5^ Patients under treatment with *BRAF* inhibitors have higher serum levels of TNFα and IFNγ and this could induce granuloma development.^6^ The literature reports that the onset of granulomatous reactions ranges between 1 and 21 months after treatment initiation, with an average range of 9 months.^7^ The most common site of involvement is usually the skin, with a reported incidence of 83%.^7^ On the contrary, in patients with systemic sarcoidosis, skin involvement occurs in only 16–32%.^8^ Papules and plaques are the most frequent clinical presentation of cutaneous SLR reactions. It can also appear as subcutaneous nodules on the arms or legs.^1^ It is noteworthy to point out that lesions can occur in areas with previous scars or chronic stimulation. Dermoscopy may orientate the diagnosis of granulomatous dermatitis, showing yellow to orange structureless areas with linear vessels.^9^

In most cases, SLR is a benign condition and potent topical steroids should be preferred for patients with only skin lesions, avoiding therapy interruption. Spontaneous resolution of the skin lesions has also been reported. In cases with severe systemic involvement, discontinuation of the target therapy or the use of systemic corticosteroids or immunosuppressants may adversely affect prognosis.^10^

Even though uncommon, the development of sarcoidosis and SLR can occur during treatment with *BRAF* inhibitors. Sarcoidosis has been described as a great simulator and may imitate metastatic disease, so it is imperative to rule out systemic involvement in order to start prompt management.

The patient provided a written informed consent form for the image and publication of case details.

## AUTHOR CONTRIBUTIONS

Maria Francesca Baracca: conceptualization, writing, editing, revision. Martina Lambertini: conceptualization, writing, editing, revision. Lidia Sacchelli: editing, revision. Cosimo Misciali: editing, revision. Barbara Melotti: editing, revision. Carlotta Gurioli: editing, revision. Emi Dika: conceptualization, writing, editing, revision.

## CONFLICT OF INTEREST

The author declares that there is no conflict of interest.

## Data Availability

The data that support the findings of this study are available from the corresponding author upon reasonable request.
